# Optimization of Fair Arterial Spin Labeling Magnetic Resonance Imaging (ASL-MRI) for Renal Perfusion Quantification in Dogs: Pilot Study

**DOI:** 10.3390/ani14121810

**Published:** 2024-06-17

**Authors:** Amber Hillaert, Luis Carlos Sanmiguel Serpa, Yangfeng Xu, Myriam Hesta, Stephanie Bogaert, Katrien Vanderperren, Pim Pullens

**Affiliations:** 1 Department of Morphology, Imaging, Orthopedics, Rehabilitation and Nutrition, Faculty of Veterinary Medicine, Ghent University, 9820 Merelbeke, Belgium; amber.hillaert@ugent.be (A.H.);; 2 Department of Medical Imaging, Ghent University Hospital, 9000 Ghent, Belgium; 3 Ghent Institute for Functional and Metabolic Imaging, Ghent University, 9000 Ghent, Belgium; 4 Department of Diagnostic Sciences, Faculty of Medicine and Health Sciences, Ghent University, 9000 Ghent, Belgium; 5 Institute of Biomedical Engineering and Technology (IBiTech)—MEDISP, Faculty of Engineering and Architecture, Ghent University, 9000 Ghent, Belgium

**Keywords:** ASL-MRI, renal blood flow, kidney, dog

## Abstract

**Simple Summary:**

Changes in renal blood flow may play an important role in the onset and progression of kidney disease. Evaluation of this parameter is of great interest because it may be reduced early in the progression of renal disease even before other indications of renal dysfunction. Non-invasive measurement of renal blood flow would greatly advance our understanding of renal disease and aid in evaluating therapeutic approaches. An imaging method that allows this and offers several advantages over other imaging methods is the magnetic resonance imaging (MRI) method, arterial spin labeling (ASL). However, ASL-MRI has not been previously used for renal perfusion assessment in dogs and parameters required for accurate quantification in this species are unknown. In this study, critical parameters for renal perfusion quantification with ASL-MRI in dogs were determined. The use of dog-specific parameters obtained from this study resulted in lower perfusion values than those obtained by using standard scanner settings. In conclusion, this study determined preliminary parameters essential for ALS-MRI-based renal blood flow quantification in dogs. These optimized parameters could provide a more reliable estimate of renal blood flow for dogs when using ASL-MRI. Further research is needed to confirm these values, but it can help guide future research.

**Abstract:**

Arterial spin labeling (ASL) MRI allows non-invasive quantification of renal blood flow (RBF) and shows great potential for renal assessment. To our knowledge, renal ASL-MRI has not previously been performed in dogs. The aim of this pilot study was to determine parameters essential for ALS-MRI-based quantification of RBF in dogs: T_1, blood_ (longitudinal relaxation time), λ (blood tissue partition coefficient) and TI (inversion time). A Beagle was scanned at 3T with a multi-TI ASL sequence, with TIs ranging from 250 to 2500 ms, to determine the optimal TI value. The T_1_ of blood for dogs was determined by scanning a blood sample with a 2D IR TSE sequence. The water content of the dog’s kidney was determined by analyzing kidney samples from four dogs with a moisture analyzer and was subsequently used to calculate λ. The optimal TI and the measured values for T_1,blood_, and *λ* were 2000 ms, 1463 ms and 0.91 mL/g, respectively. These optimized parameters for dogs resulted in lower RBF values than those obtained from inline generated RBF maps. In conclusion, this study determined preliminary parameters essential for ALS-MRI-based RBF quantification in dogs. Further research is needed to confirm these values, but it may help guide future research.

## 1. Introduction

The renal blood flow (RBF) of normal functioning kidneys is maintained within a defined range by autoregulatory mechanisms [1]. However, substantial changes in renal perfusion are known to occur with the onset and progression of both acute kidney injury (AKI) and chronic kidney disease (CKD) [2,3,4,5,6]. The ability to non-invasively measure regional renal perfusion would greatly advance our understanding of renal pathophysiology and aid in the evaluation of new therapeutic approaches. Currently, there are several techniques for evaluation of renal perfusion. However, some of these techniques, such as microspheres and implanted ultrasonic flow probes, are invasive or require post-mortem analysis [7,8,9]. Other techniques such as contrast-enhanced ultrasonography (CEUS), Doppler ultrasound, computed tomography (CT), positron emission tomography (PET) and scintigraphy have other important disadvantages including radiation exposure, relative perfusion quantification or renal toxicity of contrast agents [7,8,9]. Functional magnetic resonance imaging (fMRI), on the other hand, is an emerging imaging technique showing great potential for renal assessment [10,11].

Arterial spin labeling (ASL) is an fMRI modality that allows non-invasive quantification of tissue perfusion by generating endogenous contrast through magnetization of blood-water protons [12,13]. In ASL-MRI, radiofrequency (RF) pulses apply a label to arterial blood water protons supplying the imaging plane by inverting their longitudinal magnetization [12,13]. Image acquisition occurs after a fixed delay time, called inversion time (TI), to allow for the transit of the labeled blood into the imaging slices [12,13]. In these imaging slices, a magnetization change is induced by the labeled blood protons [12,13]. Subsequently, the acquisition is repeated without labeling to generate a control image with unaffected magnetization of the inflowing blood protons [12,13]. A perfusion-weighted image, where signal intensity reflects the local perfusion, is created by subtracting the label from the control image since the inverted magnetization of arterial blood is the only factor causing the signal difference [12,13]. The relationship between the signal difference from the perfusion weighted images and the actual blood perfusion depends on the longitudinal relaxation time (T_1_) of blood, TI and the blood-tissue water partition coefficient (λ) among others [12,13,14]. To quantify perfusion, a kinetic model is used that takes these factors into consideration [12,13,14].

The T_1_ relaxation time of blood is the time needed for the magnetization of the water protons in blood to realign with the external magnetic field after being disturbed by RF pulses [15]. Blood’s T_1_ relaxation time causes the applied label to decay [16]. The ratio of water in the renal tissue and the circulating blood is expressed with symbol λ [17]. Since the contrast agent is water-based, this ratio must be known to adjust for the distribution volume and allow accurate perfusion quantification [17]. In the literature, diverse values of λ are used to quantify renal perfusion using ASL-MRI in both humans and rodents. [18,19,20,21,22,23,24,25,26,27]. The brain λ-value is often used as it is assumed to be similar to the kidney λ-value [28]. For dogs, critical parameters for quantification of RBF with ASL-MRI such as T_1,blood_, λ and TI are unknown. The purpose of this pilot study was to optimize the TI of the FAIR ASL sequence for renal perfusion quantification in dogs. In addition, the purpose was to determine the parameters used in the kinetic model for perfusion quantification in dogs including T_1,blood_ and λ.

## 2. Materials and Methods

### 2.1. Dogs

#### 2.1.1. MRI

A healthy purpose-bred beagle was scanned (male intact, 3 years) with a body weight of 10 kg. The study was approved by the Animal Ethics Committee from the Faculty of Veterinary Medicine and the Faculty of Bioscience Engineering of Ghent University, Belgium (Approval number: EC2022-12).

#### 2.1.2. T_1_ Blood

An EDTA-anticoagulated blood sample of one dog, taken as part of the scan protocol, was used to determine the longitudinal relaxation time of blood $\left(T_{1,\mathrm{blood}}\right)$.

#### 2.1.3. Blood-Tissue Water Partition Coefficient

Kidneys were collected from client-owned dogs euthanized at the Small Animal Teaching Hospital of the Faculty of Veterinary Medicine of Ghent University (Merelbeke, Belgium). Owners’ permission was obtained to use the dog carcasses for scientific research. Kidney samples were collected between 3 and 12 h after euthanasia, with carcasses kept cooled until collection. During the selection of carcasses for sampling, the history of the dogs was checked to ensure that there were no indications of kidney disease or that the reason for euthanasia was not a condition that affected the kidneys. The reason for euthanasia was a neurological disorder in three dogs and trauma in one dog. Table 1 summarizes the details of the dogs studied.

A small tissue sample weighing from 3.58 to 7.3 5 g was excised from each kidney. After the wet weight of a kidney sample was determined on a precision balance (Ohaus^®^, Pine Brook, NJ, USA), samples were placed in a moisture analyzer (HB43 of Mettler Toledo^®^, Columbus, OH, USA) with a sample pan. The renal sample was dried at a temperature of 105 °C to a constant weight with a mean drying time of 69 min. At the end of the drying process, the tissue weight was evaluated again on a precision balance. The percentage of kidney water content was calculated according to Equation (1).

Kidney water content (%) = 100 × ((wet weight − dry weight)/wet weight)(1)

### 2.2. Anesthesia

Water and food were withheld from the dog for five and 12 h before the MRI scan. A 22-gauge IV catheter was placed in a cephalic vein and butorphanol (0.2 mg/kg) (Dolorex^®^; MSD Animal Health, Boxmeer, The Netherlands) was injected intravenously to sedate the dog. Anesthesia was induced with propofol (4–6 mg/kg IV) (PropoVet^®^; Zoetis, Louvain-la-Neuve, Belgium) in combination with midazolam (0.2 mg/kg IV) (Midazolam Accord Healthcare^®^; Accord Healthcare Limited, Whiddon Valley, UK). Anesthesia was maintained using isoflurane (Isoflutek^®^; Laboratorios Karizoo, Caldes de Montbui, Spain) 1.2–1.4% in 100% oxygen delivered via a circle rebreathing system to the dog that was intubated. An Ohmeda Veterinary Anesthesia Machine with Isotec 3 Cyprane style vaporizer (Cyprane, Keighley, UK) was used. Blood oxygen saturation and heart rate were monitored during anesthesia by pulse oximetry.

### 2.3. MRI

#### 2.3.1. Scan Protocol

The MRI examinations were performed with 3T MRI scanner (Siemens 3T Magnetom Prisma Fit, Siemens AG, Healthcare Sector, Erlangen, Germany) running VE11C with an 18 Channel Body Coil (Body 18, Siemens AG, Healthcare Sector, Erlangen, Germany).

#### 2.3.2. ASL

The FAIR Q2TIPS ASL sequence (Siemens work-in-progress research package (WIP) was used to perform ASL measurements with the following settings: imaging matrix 64 × 64, field of view (FOV) 272 mm × 136 mm, voxel size 2.1 mm × 2.1 mm × 8.0 mm, bolus length 1000 ms, TR/TE 4500/23.58 ms, flip angle 180°, bandwidth 4340 Hz/Px, 8 oblique coronal 8 mm slices.

**Figure 1:**
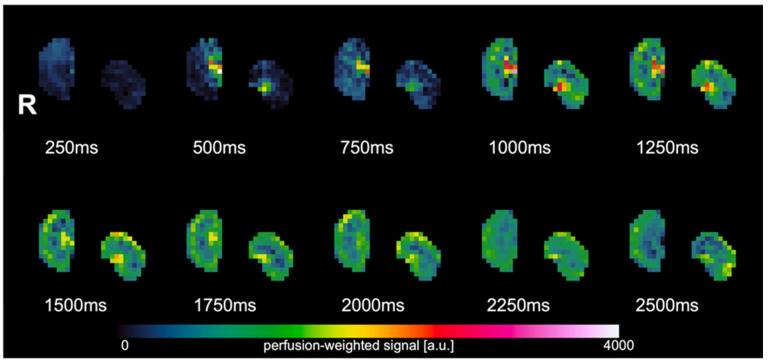
Perfusion-weighted images of the multi-TI experiment. The highest perfusion signal is observed at TI = 2000 ms.

To determine the optimal TI value, a multi-TI FAIR QTIPS ASL scan was performed first with TIs ranging from 250 to 2500 ms (250, 500, 750, 1000, 1250, 1500, 1750, 2000, 2250, 2500 ms) (Figure 1). There were 5 measurements per TI. Afterwards, a single-TI FAIR QTIPS ASL scan was performed with the optimal TI (TI = 2000 ms) and 30 measurements.

#### 2.3.3. T_1_ Blood

The blood sample was scanned with a 2D single-slice inversion recovery turbo spin echo sequence following the literature [29], TR/TE = 10,000/8.8 ms, Voxel size 0.8 mm × 0.8 mm × 5 mm, we turbo factor 7, bandwidth: 352 Hz/Px, with inversion times 50, 100, 400, 1000, 1600, 1900 ms.

### 2.4. Post-Processing

#### 2.4.1. ASL

Segmentation and statistics were conducted with FSL (https://fsl.fmrib.ox.ac.uk/, accessed on 20 September 2023). RBF maps were calculated on the scanner following Equation (2) [30]:

*f* = (λ/2αTI_2_) × (∆M(TI)/M_0_) × exp (TI_1_/T_1_) (2)

where *f* = perfusion rate (mL/100 g/min), λ = blood-tissue water partition coefficient, α = inversion efficiency, TI_1_ = inversion time, TI_2_ = bolus length, T_1_ = longitudinal relaxation time.

#### 2.4.2. T_1_ Blood

The T_1_ value was estimated by fitting the signal intensities using qMRLab—MATLAB software [31] in Matlab R2021b (version 9.11.0, The Mathworks, Natick, MA, USA).

#### 2.4.3. Blood-Tissue Water Partition Coefficient

To calculate λ, Equation (3) was used [28]:

λ = (K_w_/B_w_)/B_d_ (3)

where K_w_ is the water content of the kidney as obtained from the moisture analyzer measurements, and $B_{d}$ is the blood density (1.05 g/mL) obtained from literature [32]. The blood water content, B_w_ (g/mL), was calculated following Equation (4) [28]:

B_w_ = (Hct × d_rbc_ × w_rbc_) + [(1 − Hct) × d_pl_ × w_pl_] (4)

where Hct is the hematocrit (47.1%) [33], $d_{\mathrm{rbc}}$ is the red cell density (1.07 g/mL) [34], $w_{\mathrm{rbc}}$ is the red cells water content (0.68 g/g) [35], $d_{\mathrm{pl}}$ is the density of plasma (1.03 g/mL) [36], $w_{\mathrm{pl}}$ is the plasma water content (0.93 g/g) [35] which were obtained from the literature.

### 2.5. Statistics

Data analysis was performed in R version 4.2.2, including calculating mean and standard deviation.

## 3. Results

Figure 1 shows the FAIR Q2TIPS perfusion-weighted images (PWI) at multiple TIs ranging from 250 ms to 2500 ms. From the PWI images, the tissue difference signal (∆M) was seen to increase with increasing TI until 2000 ms, followed by a decrease at longer TI.

**Figure 2:**
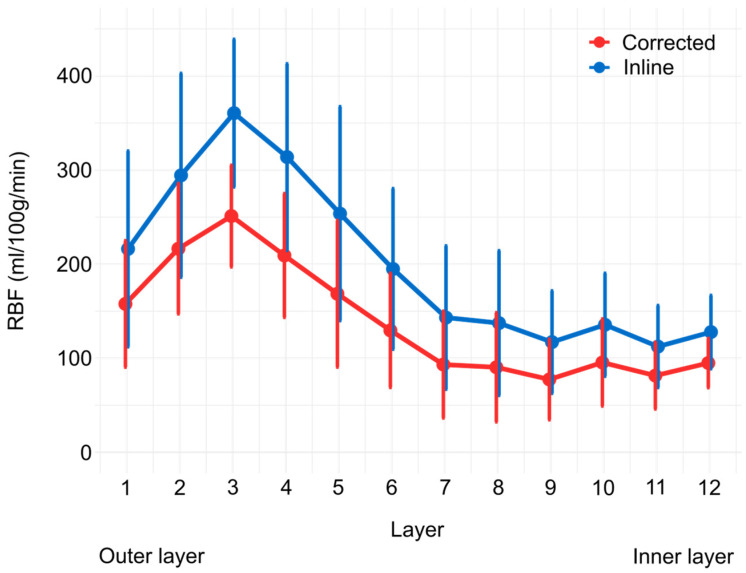
RBF plot (mean ± SD) of values obtained with the corrected RBF map (red) and inline RBF map (blue) using the TLCO method. Layer 1 represents the outermost layer and layer 12 represents the innermost layer.

The total water content of the canine kidney ranged from 79.1% to 80.7%, with mean values of 80.2%, 80.3%, 79.1% and 80% for dogs 1, 2, 3 and 4, respectively. The obtained value for $T_{1,\mathrm{blood}}$ was 1463 ms and for *λ* for water in canine kidney was 0.91 mL/g. Figure 2 illustrates the mean and SD RBF in the left and right kidney of each layer using the TLCO method [37,38] based on the corrected and inline RBF map. Table 2 summarizes the mean RBF obtained from the corrected and inline RBF map for both kidneys together using the TLCO method. The RBF values from corrected RBF maps are lower and have lower standard deviations than the RBF values from inline generated RBF maps.

## 4. Discussion

This study determined the essential parameters for renal blood flow quantification in dogs by ASL-MRI. With the FAIR ASL sequence, a TI of 2000 ms generated the best perfusion-weighted images. The measured values for the venous T_1,blood_ value and the *λ* for water in the canine kidney were 1463 ms and 0.91 mL/g, respectively. These optimized parameters for dogs resulted in lower RBF values than those obtained from inline generated RBF maps using default settings. Part of the research in this paper was presented earlier as a conference abstract [39].

The first fundamental parameter for accurate renal perfusion quantification with ASL-MRI is TI [16,40]. Due to T_1_ relaxation, the magnetization of labeled blood returns to its initial prelabeled condition within a matter of seconds [16,40]. The TI should be long enough to allow the labeled blood to arrive into the imaging slices. It should be short enough, however, to guarantee that there is enough signal for a reliable measurement before longitudinal relaxation causes all of the labels to fade [16,40]. The results of the current study indicate that the optimal TI for renal ASL-MRI in dogs is 2000 ms, which is slightly longer than the TI for humans. The TI in humans generally ranges from 1200 to 1500 ms for an ASL scan at 3T with FAIR labeling method [20,41,42,43,44]. The optimal value for TI may vary with the labeling method or scanner field strength [45].

Reliable ASL-MRI measurement of renal perfusion also requires correction for the loss of label caused by T_1_ relaxation [16,46]. The longitudinal relaxation time of the labeled spins is assumed by researchers to follow the decay of the blood T_1_ as the label is predominantly found in blood and is used in order to compensate for this loss [16,46]. Human blood T_1_ values are around 1600 ms [44,47]. The blood T_1_ of the canine sample measured in this study was 1463 ms at a typical dog Hct of 47% [33]. However, a normal physiologic hematocrit in dogs can vary over a wide range from 38 to 58% [33]. Studies have shown that variations in hematocrit can have an important effect on RBF values obtained with ASL [16,47]. The hematocrit influences the T_1_ of blood and the *λ* through its effect on the water content of blood [16,28,47]. It is therefore best to take individual variations in hematocrit into account.

There is a lot of variation in the literature when it comes to the values of *λ* that are used to quantify RBF with ASL-MRI. In humans, values ranging from 0.80 to 0.94 mL/g have been applied [18,19,20,21,22,23,24,25,26,27]. In pigs, the *λ* value even varied between 0.80 and 1 mL/g [48,49,50,51]. In many human studies [23,24,25,26] and even in animal studies (rabbits and mice) [52,53,54], the mean *λ* value of human brain tissue (0.9 mL/g) was used as an equivalent for kidney *λ* because of the assumption that these *λ* values are similar [28]. However, no comprehensive studies have been conducted to confirm this statement or clarify whether it is applicable to all species of animals. The *λ* value of the kidney and brain may differ, particularly given that the kidney has greater vascularity and that tissue water density can vary depending on the tissue type [53,55]. In our study, the *λ* of canine kidney tissue was found to be 0.91 mL/g. This value is close to the average for human brain tissue, which seems to support the general statement.

Research pertaining to the measurement of renal perfusion using ASL-MRI often presupposes that *λ* is constant throughout the renal tissue. Only one value for *λ* of water for the entire kidney was established in this investigation as well. However, using an adjusted *λ* value for each renal region may be more accurate. According to some studies, there may be regional variations in the water content of the kidneys. Research on the kidneys of rats [56] and rabbits [57] found an increasing water content from the cortex to the inner medulla. The cortex contained around 80% water, and the inner medulla around 90% [56,57]. A lack of consideration of these regional differences may lead to errors in RBF quantification. Further research is needed to determine whether a similar gradient in the kidney water content is present in the dog.

The use of dog-specific parameters for RBF quantification with ASL-MRI may provide more accurate RBF measurements in dogs. The RBF values obtained with optimized parameters for dogs were lower than those obtained with scanner-generated RBF maps. The scanner generates inline RBF maps with a fixed *λ* value of 0.90 and a user-defined T_1,blood_ of 1250 ms. Compared to the canine-specific calculated values, these standard scanner values are lower.

There are some limitations to this study. First, the study is limited by the small number of subjects and samples used to determine the different parameters. Verification of the values requires examining a greater number of samples and subjects. Considering the large differences in body size among dog breeds, it is also necessary to investigate the need for breed-specific TI values. Another limitation was determining the water content of the entire kidney instead of determining the water content of each kidney region separately. As was previously mentioned, inaccurate RBF quantification can result from neglecting these regional variations [56,57]. Therefore, it is unclear whether an adjusted *λ* value per kidney region would be necessary in dogs. A third limitation is that a venous blood sample was used to measure the T_1,blood_ level in dogs, while arterial blood samples are required for the quantification model [58]. The difference between arterial and venous blood T_1_ values is rather small, however [47]. Furthermore, previous studies have shown that venous blood T_1_ can be used for reliable RBF measurements [58]. Lastly, other parameters such as bolus length and α would need to be optimized for dogs as well.

## 5. Conclusions

In conclusion, this study defined the TI that generates optimal perfusion-weighted images for scanning dogs with a FAIR ASL sequence. Additionally, blood T_1_ and *λ*, which are essential for perfusion quantification via kinetic modeling, were established for dogs. This study suggests that applying optimized parameters for RBF calculation with ASL-MRI for dogs may prevent overestimation of RBF. The knowledge of these parameters is crucial for reliable RBF quantification with ASL-MRI in dogs and may help guide future research. Further research is needed to confirm these values and determine whether breed-related differences exist.

## Figures and Tables

**Table 1:** Overview subject characteristics.

Dog	Breed	Age	Body Weight (kg)	Sex
1	Mixed breed	3 years 9 months	42.4	Male C.
2	Bernese Mountain Dog	7 years	32	Male
3	Italian Greyhound	12 years	9	Male C.
4	Cavalier King Charles Spaniel	4 years	10.5	Female S.

C.; castrated, S.; spayed.

**Table 2:** Overview of the mean and standard deviation of RBF in each layer derived from the corrected RBF map and inline RBF map using the TLCO method.

Layer	Inline RBF (Mean ± SD)	Corrected RBF (Mean ± SD)
1	216.21 ± 104.00	157.73 ± 67.20
2	294.40 ± 108.36	216.42 ± 69.25
3	360.50 ± 78.43	251.11 ± 54.02
4	313.83 ± 99.14	209.20 ± 65.76
5	253.66 ± 113.71	168.48 ± 77.92
6	194.90 ± 85.37	129.58 ± 60.67
7	143.19 ± 76.23	93.24 ± 56.72
8	137.35 ± 76.91	90.38 ± 58.05
9	117.15 ± 54.46	77.43 ± 42.82
10	135.55 ± 54.73	95.45 ± 46.29
11	112.39 ± 43.61	81.52 ± 35.42
12	127.78 ± 38.98	94.92 ± 26.20

## Data Availability

The original contributions presented in the study are included in the article, further inquiries can be directed to the corresponding author.
